# Potency of lactic acid bacteria isolated from balinese bovine (*Bos sondaicus*) intestinal waste from slaughterhouse to improve nutrient content of wheat pollard as animal feedstuff by fermentation process

**DOI:** 10.14202/vetworld.2018.1127-1134

**Published:** 2018-08-16

**Authors:** Widya Paramita Lokapirnasari, Adriana Monica Sahidu, Koesnoto Soepranianondo, Agus Supriyanto, Andreas Berny Yulianto, Anam Al Arif

**Affiliations:** 1 Department of Animal Husbandry, Faculty of Veterinary Medicine, Jl. Mulyorejo, Kampus C, Universitas Airlangga, Surabaya, Indonesia; 2 Department of Marine, Faculty of Fisheries and Marine, Jl. Mulyorejo, Kampus C, Universitas Airlangga, Surabaya, Indonesia; 3 Department of Biology, Faculty of Science and Technology, Jl. Mulyorejo, Kampus C, Universitas Airlangga, Surabaya, Indonesia; 4 Doctoral of Veterinary Science, Faculty of Veterinary Medicine, Jl. Mulyorejo, Campus C, Universitas Airlangga, Surabaya, Indonesia

**Keywords:** *Escherichia coli*, *Lactobacillus casei*, probiotics, *Staphylococcus aureus*, wheat pollard

## Abstract

**Aim:**

The purpose of this study was to know the genetic and biochemical identification of isolated lactic acid bacteria (LAB) from Balinese bovine (*Bos sondaicus*) intestinal waste, acidity, and ox bile salts and to inhibit the growth pathogen of *Staphylococcus aureus* and *Escherichia coli* and the potential of those isolated to improve nutrient value of wheat pollard as animal feed ingredient by fermentation process.

**Materials and Methods:**

This research was divided into three stages. The first stage, isolated LAB were obtained from the bovine intestines at a slaughterhouse in Indonesia. Small intestinal samples were collected from 10 healthy Balinese beef cattle (*B. sondaicus*). The isolated LAB were identified by VITEK 2, polymerase chain reaction, and 16S rDNA. The basic local alignment search tool (BLAST) was performed to determine the phylogenetic tree. The second stage, the LAB were screened for their tolerance at pH 2, 3, and 4; bile salt, and antagonistic to enteric pathogen. In the third stage, to determine the potency of this isolate to increase nutrient content of wheat pollard by facultative anaerobe fermentation for 3 and 5 days.

**Results:**

The result of the first stage showed that the isolate could be identified as *Lactobacillus* *casei* WPL 315. The result of the second stage showed that the isolate tolerance to low pH (pH 2, pH 3, and pH4) for 90 min and 24 h, and this isolate had viability tolerance in 0.3% bile salt. The isolate can inhibit *S. aureus* and *E. coli*. The result of the third stage by proximate analysis showed that crude protein increased by 23.08% after fermentation, while crude fiber decreased by 61.24% on the level 0.5% *L. casei* subsp. WPL 315 in the 3-day fermentation.

**Conclusion:**

Based on the results, it showed that *L. casei* WPL 315 derived from indigenous intestinal Balinese beef cattle (*B. sondaicus*) has tolerant characteristic on acidity and ox bile salts, has antagonistic effect against *E. coli* and *S. aureus*, and has the ability to increase crude protein and decrease crude fiber content of wheat pollard. It would be interesting to determine whether the strain has a probiotic candidate.

## Introduction

Feeding cost is the biggest component in the production cost of the poultry industry. To decrease feeding costs, some efforts have been taken by poultry farmers such as the addition of feed additive. The addition of various feed additives to the poultries has an important role in stimulating growth and decreasing number of feed conversion that can give positive effect on chicken growth [1]. Probiotic is one of the feed additives that have been recently developed in the poultry industries, non-pathogen living organism that has mechanism to preserve microbiota balance in the digestive tract by influencing gastric microbiota as well as eliminating microorganism of host-pathogen by creating an inconvenient atmosphere for pathogenic bacterial growth [2]. The most common microorganism species used as probiotics are *Lactobacillus*, *Bifidobacterium* [3,4] *Lactococcus, Leuconostoc*, *Enterococcus*, and *Carnobacterium* [5], *Lactobacillus acidophilus*, *Lactobacillus sporogenes*, *Lactobacillus* *plantarum*, *Lactobacillus* *rhamnosus*, *Lactobacillus* *delbrueckii*, *Lactobacillus* *reuteri*, *Lactobacillus* *fermentum*, *Lactococcus lactis*, Lactobacillus cellobiosus, *Lactobacillus* brevis, and *Lactobacillus* *casei* [6].

Probiotic ability can be explained through various mechanisms. The microorganism can produce an antimicrobial substance, compete, and colonize in the gastrointestinal tract [7,8]. Probiotic can modulate immune cells. Probiotic is directly taken up through transcytosis by microfold epithelial cells and engulfed by macrophages or dendritic cells, which eventually triggers an immune response. Cytokines modulate the immune functions of dendritic, T and B cells [9]. Probiotic has a role in increased feed consumption for most livestock. The condition is caused by the increasing of feed digestibility in an animal that causes digestive tract that can be emptied soon so feed efficiency can be achieved. Probiotic not only increases feed consumption but also promotes growth so that it can enhance the feed conversion [10].

The use of other alternative feed ingredient from agricultural by-products (i.e., wheat pollard, rice bran, and maize bran) or agricultural wastes (i.e., S rice straws, maize straws, maize leaf, and sugarcane leaves) was needed to maintain the availability of feed supply. Agricultural by-products or agricultural wastes that are available all years have low crude protein and high crude fiber content [11], such as rice bran, wheat pollard, cotton, and tofu wastes. Wheat pollard is agricultural by-products that are mostly used in livestock feeding because it is easy to get and costs lower. The limitation of wheat pollard utilization as the mixture in the livestock feed because of its low protein content, high crude fiber, and low digestibility.

To increase the feed quality based on exploration of indigenous lactic acid bacteria (LAB) from bovine intestine in the slaughterhouse, we isolated and identified LAB on acidity survival, ox bile salts survival, and to inhibit the growth of pathogen *Staphylococcus aureus* and *Escherichia coli* and the potential of the isolate, and to improve nutrient value of wheat pollard as animal feed ingredient by fermentation process.

## Materials and Methods

### Ethical approval

The research does not need ethical approval. However, samples were collected as per standard collection methods without any harm or stress to the animals.

### Research procedure

This research was divided into three stage. In the first stage, LAB isolate was obtained from the bovine intestines which were identified by VITEK 2, polymerase chain reaction (PCR), and nucleotide sequencing of 16S rDNA by comparing them to the GenBank database. The basic local alignment search tool (BLAST) was performed to determine the kinship arrangement based on the phylogenetic tree. This research only determined one species of *Lactobacillus*. In the second stage, the LAB isolate was screened further for their tolerance to low pH, at pH 2, 3, and 4 as well as ox bile salts tolerance. In the third stage, to know further the ability of this isolate to animal feed ingredient (wheat pollard) conducted fermentation process was conducted on facultative anaerobe condition for 3 days and 5 days.

### First stage

#### Genotypic identification

DNA amplification with PCR and identifying coding genes based on nucleotide sequence of 16S rDNA genomes.

Isolation of strain from the small intestine of bovine

Small intestinal samples were collected from 10 healthy Balinese beef cattle from a slaughterhouse in Indonesia. All samples were cultivated using a modified de Man Rogosa and Sharp (MRS) broth and agar. Bacterial colonies which showed clear zone surrounding their colonies were selected to biochemical identification by VITEK 2, PCR, and 16S rDNA, and a further test of basic probiotic properties including acid and ox bile salts tolerance assay, and antagonistic to enteric pathogen.

#### DNA isolation

Ingredients used in the DNA isolation process were as follows: Lysozyme 10 mg/mL, buffer TE 50 mM (50 mMtris Cl [pH 8.0]; 50 mM EDTA), buffer STEP (sodium dodecyl sulfate 0.5%, 50 mMtris Cl [pH 8.0], 0.4 M EDTA, and proteinase K), Na-acetate 3M, Phenol: chloroform:isoamyl alcohol (25:24:1), ethanol 70%, cold absolute ethanol, and distilled water.

Ingredients used in the 16S rDNA gene amplification were buffer 2.5 µl, dNTP 2.0 µl, MgSO_4_ 1.0 µl, DNA template 2.0 µl, primer forward PB 36 (10 pmol) 1.0 µl, primer reverse PB 38 (ρmol) 1.0 µl, distilled water 10.3 µl, enzyme high fidelity Taq polymerase 0.2 µl, and PCR product detection with electrophoresis: Buffer TBE (tris base/boric acid/EDTA) 0.5×, agarose, and ethidium bromide. DNA isolation was performed using Ausubel methods [12].

#### DNA Amplification with PCR

High fidelity platinum Taq DNA polymerase (Invitrogen™ Platinum™ *Taq* DNA Polymerase High Fidelity, US) kit with primer forward PB36 5’-AGR GTT TGA TCM TGG CTC AG-3’ (Invitrogen) and primer reverse PB38 5’-GMT ACC TTG TTA CGA CTT-3’ (Invitrogen) that produced ± 1400pb were used for PCR.

Master mix of used amplification reaction was 10× high fidelity PCR buffer 2.5 ml, 10 mM dNTP mix 2 ml, 50 mM MgSO_4_ 1 ml, primer forward 1 ml (10 pmol/µl), primer reverse 1 ml (10 pmol/µl), template cDNA 2 ml, platinum tag high fidelity 0.2 ml, and distilled water until it reached total volume of 20 ml. The used PCR condition was pre-denaturation at 95°C for 5 min, denaturation at 95°C for 1 min, annealing at 50°C for 1 min, extension at 72°C for 1 min, 30 cycles, and a final extension at 72°C for 10 min. PCR result was analyzed by electrophoresis gel on 2% of an agarose gel that contains ethidium bromide. 5 µl DNA added with 2 µl loading dye was added into agarose holes, and then run in the 100-volt tension for more or less 30 min.

#### Analysis of DNA sequence coding 16S rDNA

DNA sequencing coding 16S rDNA was performed by 1^st^ Base Serdang, Malaysia. Analysis of sequencing result was performed through BLAST nucleotide sequencing from 16S rDNA sequencing result with the available database on www.ncbi.nlm.nih.gov.

#### Biochemical identification

Biochemical identification by VITEK 2 microbial identification system version: 05.01 (BioMérieux) was applied in examining WPL 315 isolates. The VITEK 2 system (bioMérieux) is an integrated modular system that consists of a filling-sealer unit, a reader-incubator, a computer control module, a data terminal, and a multicopy printer. The system detects bacterial growth and metabolic changes in the microwells of thin plastic cards using a fluorescence-based technology. Different microwell cards contain biochemical substrates [13].

### Second stage

#### LAB survival test on acidity and survival test on ox bile salts, antagonistic test, on enteric pathogen bacteria

Ingredients used in this research included an antagonistic test on enteric pathogen microbe used in MRSB/de MRS Broth (Oxoid) media, nutrient agar media (NA and Oxoid), and nutrient broth (NB and Oxoid). Media used in the survival test on acidity were MRSB (Oxoid), MRSA (Oxoid), 0.85% of sterile NaCl, as well as HCl. Media used in the bile salts test were MRSB (Oxoid), MRSA (Oxoid), 0.85% of sterile NaCl, as well as ox gall 0.3% (Oxoid). Media used to test crude protein proximate analysis were Tablet Kjeldhal (Merck), H_2_SO_4_ (Merck), NaOH 40% (Merck), boric acid (Merck), methyl red (Merck) indicator, Brom cresol green (Merck), H_2_SO_4_ 0.01 N (Merck), and Aquadest.

#### Selection of LAB as a probiotic candidate

The isolate assumed to have the ability as probiotic was selected through various tests, so superior isolate of LAB was chosen to be tested *in vitro*. The tests were as follows.

#### LAB survival test on acidity and survival test on ox bile salts

Acid tolerance was assayed as reported by Succi *et al*. with modification [14], in 10 mL of MRS broth adjusted to pH values of 2.0, 3.0, and 4.0 with 3.0 M HCl. MRS broth at pH 7 served as control. All tests were carried out in duplicate.

The modification method of Gilliland and Kim [15] was employed in this study to know the effects of ox bile salts 0.3% (w/v) (Sigma, Milan, Italy) in MRS broth. All the samples were incubated at 37°C, 24 h. The aliquots were 10-fold diluted and viable bacteria (CFU/mL) were enumerated by spot plating on MRS agar (48 h, 37°C, and anaerobic conditions) [16].

#### Antagonistic test on enteric pathogen bacteria

The antagonistic test was assayed as reported by Jin *et al*. [17] with modification. Antagonistic test on enteric pathogen was performed with an agar diffusion method with modification in the pouring of pathogenic bacteria culture. LAB culture was grown on MRSB medium at 37°C for 18-20 h. After that, pathogenic bacteria were inoculated as much as 1 ose in the NB media, to be incubated for 24 h at 37°C. After incubation ended, 0.2 mL of the incubated bacteria was taken and placed into 100 mL NA media (0.2%) to be mixed well (homogeneous), and then placed into Petri dish with 1-20 mL for each dish until solid. After agar media became solid, a hole was created in the agar media with 6 mm diameter. Five holes were created for each Petri dish.

LAB culture from MRSB was spotted into the hole as much as 50 µl and then incubated for 24 h at 37°C. MRSB medium without LAB was used as the control. The observation was performed by measuring the clear zone around the hole using Vernier calipers. LAB antagonistic activity on enteric pathogen was shown as the diameter of created clear zone.

### Third Stage: Potency of L. casei WPL 315 on fermented wheat pollard

#### Inoculation of L. casei WPL 315 on fermented wheat pollard

To know isolate ability on the nutritional content changes of crude protein and crude fiber, fermentation process was performed through following treatment P0: 100 g of wheat pollard without *L. casei* WPL 315 + molasses 4% addition (as control), treatment P1: 100 g of wheat pollard with addition of 0.5% *L. casei* WPL 315 + molasses 4% (3-day fermentation), treatment P2: 100 g of wheat pollard with addition of 1.0% *L. casei* WPL 315 + molasses 4% (3-day fermentation), treatment P3: 100 g of wheat pollard with addition of 0.5% *L. casei* WPL 315 + molasses 4% (5-day fermentation), and treatment P4: 100 g of wheat pollard with addition of 1.0% *L. casei* WPL 315 + molasses 4% (5-day fermentation). The fermentation process was done in anaerobe condition. The experimental design used in this research was a completely randomized design in triplicate for each treatment. The molasses was mixed with Aquadest as much as 20% from sample weight, and the isolate based on treatment level was poured in the mixture (molasses + Aquadest) and then sprayed evenly on the wheat pollard. The mixture was then fermented in anaerobe condition in the plastic bag for 3 days and 5 days. The isolate concentration of *L. casei* WPL 315 used in this research was 1.2×10^8^ CFU/mL. After incubation ended, it was dried and continued into the proximate analysis of crude fiber and crude protein according to AOAC [18].

### Statistical analysis

Data were analyzed using one-way analysis of variance (ANOVA). If the significant differences were found, the test would be continued using Duncan’s multiple range test on 5% significance level.

## Results

### Genotypic and biochemical identification

#### DNA amplification with PCR and identifying coding genes based on nucleotide sequence of 16S rDNA genomes

In this research, a colony of WPL 315 LAB was capable of growing on MRSA medium. Based on Gram staining, this LAB isolate was Gram-positive (GP), rod-shaped, and positive motility.

An advanced test was conducted on code WPL 315 LAB isolate with 16S rDNA and phylogenetic tree structure with 91-98% similarity. The majority of bacteria resembling WPL 315 isolate originated from *Lactobacillus* genus. Based on the degree of similarity of nucleotide structure, the closeness in position with *L. casei* ATCC 334 (accession NC_008526.1; 98% identity, Table-1), and inherited traits in congruence with microbe identification system, the isolated strain was identified as *L. casei* WPL 315.

**Table-1:** Similarity identity

Description	Identities	Accession (sequence ID):
*Lactobacillus casei* ATCC 334 chromosome, complete genome	98%	ref|NC_008526.1
*Lactobacillus rhamnosus* GG whole genome sequence, strain GG (ATCC 53103)	98%	ref|NC_013198.1
*Lactobacillus sakei strain* 23K complete genome	94%	ref|NC_007576.1
*Pediococcus claussenii* ATCC BAA344, complete genome	94%	ref|NC_016605.1
*Pediococcus pentosaceus* ATCC 25745, complete genome	94%	ref|NC_008525.1
*Lactobacillus buchneri* CD034, complete genome	92%	ref|NC_018610.1
*Lactobacillus reuteri* DSM 20016, complete genome	92%	ref|NC_009513.1
*Lactobacillus plantarum* WCFS1, complete genome	92%	ref|NC_004567.2
*Lactobacillus fermentum* IFO 3956 DNA, complete genome	92%	ref|NC_010610.1
*Lactobacillus brevisATCC* 367, complete genome	91%	ref|NC_008497.1

Biochemical assay of LAB isolate WPL 315 was investigated using the VITEK 2 Compact system. The GP card of the VITEK 2 system includes biochemical tests to determine carbohydrate usage, enzyme activity, and resistance to certain compounds that can be used to identify GP, non-spore-forming bacteria [19]. The result of phenotypic identification of LAB isolates WPL 315 by VITEK 2 microbial identification system *version*: 05.01 (BioMérieux) as shown in Table-2.

**Table-2:** Biochemical test of LAB isolate WPL 315 by VITEK 2

Biochemical test	Reaction	Biochemical test	Reaction
LAC (Lactose)	+	dRIB (d-Ribose)	+
SAC (Saccharose/Sucrose) Sucrose	+	dGAL (D-Galactose) Galactose	+
Gluconate	+	Celobiose	+
dRIB (D-Rybosa) (Ribose)	+	dRAF (D-Raffinose) Raffinose	−
dXYL (D-Xylose) Xylose	−	Mannitol	+
ARG (Arginin)	−	Ramnose	−
Arabinose	−	Esculin	+
BXYL (beta-xylosidase)	+	LeU (leucinearylamidase)	+
BGAL (beta-galactosidase)	+	AlaA (alanine arylamidase)	+
APPA (Ala-Phe-Pro Arylamidase)	+	GLYG (Glycogene)	−
ELLM (Ellman)	−	MTE (Maltotriose)	−
dMNE (D-Mannose)	+	PLE (Palatinose)	−
BMAN (Beta-mannosidase)	−	AGLU (alpha-glucosidase)	+
INU (Inulin)	−	PSCNa (putrescine assimilation)	−
OLD (oleandomycin resistance)	+	POLYB_R (Polymixin_B resistance)	+
LysA (L-lysine Arylamidase)	+	PheA (phenylalanine arylamidase)	+
PyrA (L-pyrrolidonylarylamidase)	+	TyrA (Tyrosine Arylamidase)	+
CDEX (cyclodextrin)	−	INO (Inositol)	−
MdX (Methyldxyloside)	−	GlyA (glycine arylamidase)	−
dMLZ (D-melezitose)	−	IRHA (L-rhamnose)	+
PHC (phosphoryl choline)	−	dTAG (dTagatose)	+
dGLU (D-glucose)	+	NaCl 6.5% (growth in 6.5% NaCl)	+
ESC (esculin hydrolyze)	+	ProA (L-prolinearylamidase)	+
AspA (L-aspartate arylamidase)	−	BNAG (beta-N-acetyl-glucosaminidase)	+
AGAL (alphagalactosidase)	+	MdG (methyl-A-D Glucopyranoside acidification)	−
dGAL (D-Galactose)	+	dMAN (D-Mannitol)	+
AMAN (alpha-mannosidase)	−	BGLU (beta-glucosidase)	−
NAG (N-acetyl-Dglucosamine)	+	dTRE (D-Trehalose)	+
PVATE (pyruvate)	+	KAN (kanamycin resistance)	+
TTZ (tetrazolium red)	+

LAB=Lactic acid bacteria

#### LAB survival test on acidity

In the digestive tract, bactericidal effect from acid happened at pH under 2.5 [20]. The result of survival test on acidity showed that *L. cassei* WPL 315 tolerance to low pH (Table-3).

**Table-3:** LAB survival test on acidity

Survival test on acidity of *L. casei* WPL 315				

Time	MRS agar (control) (CFU/mL)	MRS agar pH 2 (CFU/mL)	MRS agar pH 3 (CFU/mL)	MRS agar pH 4 (CFU/mL)
90 (min)	2.90×10^8^	6.20×10^7^	1.50×10^8^	2.60×10^8^
Duplicate	3.00×10^8^	6.20×10^7^	2.40×10^8^	2.90×10^8^
24 (h)	1.10×10^9^	1.00×10^7^	2.00×10^7^	2.25×10^8^
Duplicate	1.20×10^9^	1.00×10^7^	2.00×10^7^	2.40×10^8^

LAB=Lactic acid bacteria, *L. casei=Lactobacillus casei*

#### Survival test on ox bile salts

The results of this research showed that *L. casei* WPL 315 has viability tolerance in ox bile salts 0.3% and the concentration of *L. casei* WPL 315 in ox bile salts 0.3% (Table-4).

**Table-4:** LAB survival test on oxbile salts after 24 h, starting inoculums 2.90×10^8^

Lactide acid bacteria viability isolate (ox bile tolerance 0.3%)	Isolate
9.6×10^7^ CFU/ml	*L. casei* WPL 315

LAB=Lactic acid bacteria, *L. casei=Lactobacillus casei*

#### Antagonistic test on enteric pathogen bacteria

The result of the antagonistic test on enteric bacteria shows that *L. casei* WPL 315 has an antagonistic effect against *E. coli* and *S. aureus*. The index antibacterial is shown in Table-5.

**Table-5:** LAB survival test on *E. coli* and *S. aureus*

Antagonistic test on enteric bacteria	Diameter inhibition (mm)
*E. coli*	2.0
*S. aureus*	1.5

LAB=Lactic acid bacteria, *L. casei=Lactobacillus casei, E. coli=Escherichia coli, S. aureus=Staphylococcus aureus*

#### Inoculation of L. casei WPL 315 on fermented wheat pollard

The result of statistical analysis using one-way ANOVA showed that the use of *L. casei* WPL 315 on wheat pollard fermentation had a significant effect in the pH, crude protein, and crude fiber content of wheat pollard (p<0.05). The result of wheat pollard fermentation showed the decreasing of crude fiber content and the increasing of crude protein content at 0.5% *L. casei* WPL 315 isolate within 3-5-day fermentation as shown in Table-6.

**Table-6:** Analysis result on nutrient content changes in wheat pollard fermentation using *L. casei*WPL 315 isolate

Treatment	pH	Crude protein	Crude fiber
P0 (control)	7^a^±0.11	13.0^a^±0.21	12.9^a^±0.21
P1 (0.5%, 3 days)	5^b^±0.13	16.3^c^±0.18	5.0^c^±0.18
P2 (1.0%, 3 days)	5^b^±0.10	14.0^ab^±0.45	8.2^ab^±0.45
P3 (0.5%, 5 days)	5^b^±0.10	15.0^b^±0.19	6.0^b^±0.19
P4 (1.0%, 5 days)	5^b^±0.13	14.5^ab^±0.47	8.0^ab^±0.47

^a,b,c^Means in the same column with the different superscript are significantly different at (p≤0.05). *L. casei=Lactobacillus casei*

## Discussion

### Genotypic and phenotypic identification

#### DNA amplification with PCR and identifying coding genes based on nucleotide sequence of 16S rDNA genomes

To identify and determine the taxonomy of bacteria from several environment sources and identify the phylogenetic characterization, 16S rDNA gene sequencing can be applied since this molecule exists in every organism with identical function in all organisms [21-23]. The BLAST nucleotide (BLASTn) program (available at http://blast.ncbi.nlm.nih.gov) was used to screen candidate genes based on sequence similarity [24]. An advanced assay was conducted on code WPL 315 LAB isolate with 16S rDNA and phylogenetic tree structure with 91-98% similarity. The majority of bacteria resembling WPL 315 isolate originated from *Lactobacillus* genus. Based on the degree of similarity of nucleotide structure, the closeness in position with *L. casei* ATCC 334 (accession NC_008526.1; 98% identity, Table-1), and inherited traits in congruence with microbe identification system, the isolated strain was identified as *L. casei* WPL 315.

VITEK2 Compact (bioMerieux, France) is an automated system able to identify microorganisms by testing 59 biochemical properties and also handle many samples in one reaction. VITEK2 compact was used for this study to differentiate isolates at a strain level by analyzing and comparing the phenotypes. Strains were individually grown on MRS agar. Colonies were picked and mixed in a 0.45% NaCl solution until the McFarland standard measured 0.50-0.63 on the VITEK 2 DensiCheck instrument (bioMerieux). GP colorimetric identification cards (bioMerieux) and the tubes containing the bacteria were assembled in a cassette and assayed using the VITEK2 compact system. Data were analyzed using the VITEK 2 software version VT2-R03.1.

LAB isolated from the intestine of local beef cattle produced several enzymatic activities: Beta-xylosidase, beta-galactosidase, Ala-Phe-Pro Arylamidase, l-lysine arylamidase, l-pyrrolidonyl arylamidase, alpha-galactosidase, leucine arylamidase, alanine arylamidase, alpha-glucosidase, phenylalanine arylamidase, tyrosine arylamidase, and beta-n-acetylglucosaminidase. The proteolytic system of LAB is composed of a cell envelope-associated proteinase, peptide transport systems, and intracellular peptidases. It can hydrolyze proteins to small peptides and amino acids which are essential for rapid microbial growth [25].

β-glucosidases enzymes are responsible for the catalyze of β-1,4-glycosidic bonds of various oligosaccharides, disaccharides, and alkyl- and aryl-β-d-glucosides [26], responsible for the hydrolysis of ce-lo-oligosaccharides and cellobiose, an important fiber source in cereal feeds. In addition, these enzymes hydrolyze toxic and/or bitter glucosides, release aromatic compounds, and synthesize various oligosaccharides, glycoconjugates, and alkyl- and amino-glucosides [27].

#### LAB survival test on acidity

The result of survival test on acidity showed that *L.cassei* WPL 315 tolerance to low pH (Table-3). This was comparable with *L. casei* IS-7257 has viability as much as 5.22±0.31 log CFU/mL. The survival on acid tolerance indicated the ability of the isolate to survive in stomach that has extreme pH (pH 2) and could survive in the gastrointestinal tract process where hydrolytic and gastric juice are secreted [28].

These results are in agreement with those obtained from previous similar studies, where *Lactobacillus* strains were able to survive when exposed by pH 2.5-4.0 but displayed loss of viability at lower pH values [29,30]. Lactic acid produced by *Lactobacillus* creates an acid environment that can inhibit the growth of pathogenic bacteria [31]. Other research showed that some LAB strains have function as competitive inhibitors on pathogenic organism [32], the strains include L. casei 99p, L. *rhamnosus* GG, L. *casei* Shirota, *Bifidobacterium breve* Yacult, and L. *acidophilus* [33].

#### Survival test on bile salts

Bile tolerance and acid tolerance are required for bacterial growth in the small intestine and survive passage through the stomach. The result of this research shows that *L. casei* WPL 315 has viability tolerance in 0.3% bile salts. The results showed that the concentration of *L. casei* WPL 315 in 0.3% bile salts was 9.6×10^7^ CFU/mL in MRS agar (Table-4). Similar observations were also reported by Srinu *et al*. [34] and Balasingham *et al*. [35] that LAB strains survived and tolerated at 0.3-2.0% bile salts (Oxgall). The viability tolerance in the bile salts condition is one of the main criteria for *in vitro* selection of potentially probiotic bacteria and microbes [36]. Because the bacterial cell wall is comprised mainly of phospholipids, bile salts which are an emulsifier and solubilizes the lipid that can damage the bacterial cells [37].

#### Antagonistic test on enteric pathogen bacteria

Inhibition of pathogens by the intestinal microbiota has been called bacterial antagonism, bacterial interference, barrier effect, colonization resistance, and competitive exclusion. Mechanisms by which the indigenous intestinal bacteria inhibit pathogens include competition for colonization sites, competition for nutrients, production of toxic compounds, or stimulation of the immune system [38]. LAB strains have potency in creating bactericidal bioactive peptides. Bacteriocins are also produced by species from *Lactobacillus*, L. acidophilus produces lactacin B or F, and L. *casei* B80 produces casein 80 [39,40]. Antimicrobial activity produced by LAB strain is not correlated with the acidity level in the medium. It has been reported that LAB strain has a strong inhibitory effect on *S. aureus* growth in milk. The inhibition ability is correlated with the existence of bacteriocins production, hydrogen peroxide production, and organic acids production such as lactic acid and acetic acid [41,42].

#### Inoculation of L. casei WPL 315 on fermented wheat pollard

The result of wheat pollard fermentation showed the increase of nutrient that was shown by the decreasing of crude fiber content and the increasing of crude protein content at 0.5% level within 3-5-day fermentation as shown in Table-6. The result of the statistical analysis showed that the use of *L. casei* WPL 315 on wheat pollard fermentation had a significant effect in the content of pH wheat pollard (p<0.05). The result of pH level analysis showed the decrease of pH within the incubation process for all treatment groups compared to that in the control group (P0). *L. casei* WPL 315 treatment showed that the lowest pH was achieved in the treatment that used 0.5% isolate addition in the fermentation process because it was caused by LAB activity in recasting activity on water-soluble carbohydrate contained in the wheat pollard in the form of lactic acid. The decrease in pH level was followed by the decrease in carbohydrate level.

The result of the statistical analysis showed that the use of *L. casei* WPL 315 on wheat pollard fermentation had a significant effect on the content of crude fiber in wheat pollard (p<0.05). The result of the analysis showed a decreasing level of crude fiber content for all treatment compared to that in the control group (P0). The lowest crude fiber content was achieved in the treatment group that used 0.5% isolate within 3 days’ fermentation process. The decrease of crude fiber content was correlated with the isolate ability to degrade organic matter derived from complex molecules becoming simplest molecules: Cellulose was degraded into cello-oligosaccharide to be then degraded into cellobiose; in the end, cellobiose was degraded into glucose [6]. Probiotics also stimulate activities of cellulolytic bacteria to degrade crude fiber [10].

The result of the statistical analysis showed that the use of *L. casei* WPL 315 on wheat pollard fermentation had a significant effect on crude protein content (p<0.05). The result showed an increasing level of crude protein content for all treatment compared to the control group (P0). The highest crude protein content was achieved by adding 0.5% isolate within 3-day fermentation. This was caused by increased activity of *L. casei* WPL 315 in binding N as the basic matter to synthesize protein. Thus, the increase of nitrogen level allowed bacteria to grow and perform activity optimally that made crude protein level in wheat pollard increased higher compared to that in other treatment groups because bacteria are a single cell protein. The increase of crude protein content was also caused by the decrease of other compounds including nitrogen-free extract produced by fermented crude fiber [4]. Enzyme β galactosidase, glycols, and lactate dehydrogenase could be produced by LAB. It has a role in decreasing pH in the gastrointestinal tract, so it will inhibit *E. coli* growth and other pathogenic bacteria that need pH 6-7 [20].

## Conclusion

The result of the research showed that *L. casei* WPL 315 derived from indigenous intestinal Balinese beef cattle (*Bos sondaicus*) has tolerant characteristic on acidity and ox bile salts and has antagonistic effect against *E. coli* and *S. aureus*.

## Authors’ Contributions

The work was done by WPL who designed the research and WPL, AS, as well as ABY who conducted the experimental work. WPL, AMS, and AA analyzed and interpreted the data and drafted the manuscript. WPL, KS, and AA participated in doing data collection, data analysis, data interpretation, and writing the manuscript. All authors read and approved the final manuscript.
